# Association of Concurrent Amiodarone and Beta-Blocker Therapy at Discharge With 30-Day Rehospitalization After Atrial Fibrillation Hospitalization

**DOI:** 10.7759/cureus.109094

**Published:** 2026-05-18

**Authors:** Teddy A Teddy, Spencer Cadet, Edidiong Okon-Ben, Fraol T Erega, Abdelwahab Ahmed, Siri Vummaneni, Mustafa Marzoug, Kendall Bell

**Affiliations:** 1 Internal Medicine, Detroit Medical Center, Wayne State University, Detroit, USA; 2 Internal Medicine, HCA Healthcare, University of Central Florida Fort Walton Beach Hospital, Florida, USA; 3 Cardiology, Detroit Medical Center, Wayne State University, Detroit, USA

**Keywords:** 30‑day readmission, amiodarone, antiarrhythmic drugs, atrial fibrillation, beta blockers, pharmacotherapy, propensity score matching, rate control, rehospitalization, rhythm control

## Abstract

Background

Atrial fibrillation (AF) is associated with high early rehospitalization rates following index hospitalization. While beta-blockers remain the cornerstone of rate control, amiodarone is frequently used for rhythm control in selected patients. The clinical impact of concurrent amiodarone and beta-blocker therapy at discharge on short-term rehospitalization remains incompletely defined.

Methods

A retrospective cohort study using the TriNetX platform was conducted. Adult patients hospitalized with AF were identified and stratified based on discharge prescription of amiodarone plus a beta-blocker versus comparator therapy. Strict exclusion criteria were applied to minimize confounding, including exclusion of patients with recent cardiac surgery, implantable device placement, advanced conduction disease, or incomplete follow-up. Propensity score matching in a 1:1 ratio was performed using demographic and clinical variables. The primary outcome was 30-day all-cause rehospitalization. Secondary outcomes included AF-related rehospitalization, rhythm-related interventions, and safety endpoints.

Results

A total of 2,647 patients met the inclusion criteria. After propensity score matching, 1,323 patients per group were successfully matched, yielding a final analytic sample of 2,646 patients; one patient could not be matched and was excluded. Baseline characteristics were well balanced, with standardized mean differences below 0.10. At 30 days, all-cause rehospitalization occurred in 13.6% of patients receiving combination therapy compared with 16.9% in the comparator group, yielding an odds ratio of 0.77, with a 95% CI of 0.62 to 0.95 and p = 0.014. Atrial fibrillation-related rehospitalization was significantly lower in the combination group, at 5.1% versus 7.4%, with an odds ratio of 0.67 and p = 0.008. Rhythm-related urgent visits occurred in 3.6% versus 5.4%, with an odds ratio of 0.65 and p = 0.019. No significant difference in 30-day mortality was observed, at 0.9% versus 1.2%, with p = 0.40. Safety analysis demonstrated a higher rate of bradycardia- or hypotension-related encounters in the combination group, at 2.7% versus 1.5%, with an odds ratio of 1.83 and p = 0.038, without differences in pacemaker implantation.

Conclusion

Among patients hospitalized with AF, discharge on amiodarone plus beta-blocker therapy was associated with a modest reduction in 30-day rehospitalization, driven primarily by fewer rhythm-related events, with a small increase in bradycardia-related complications. These findings support a selective rhythm-control strategy in appropriately chosen patients.

## Introduction

Atrial fibrillation (AF) affects 6.1 million adults in the United States, with prevalence projected to reach 12.1 million by 2030. It accounts for over 3 million ED visits annually, with a hospitalization rate of 48.4%, and early unplanned rehospitalization rates among patients with AF and comorbidities reaching 16.0% within 30 days of discharge [1]. Despite advances in acute management, the period immediately after discharge carries elevated risks for recurrent symptoms, medication adjustments, and arrhythmia burden that frequently drive unscheduled returns to hospital care. Beta-blockers have long served as first-line therapy for rate control in AF, reducing the ventricular response and improving symptoms without directly suppressing the arrhythmia substrate, as reflected in updated guideline recommendations emphasizing early rhythm control and the role of beta-blockers as first-line agents for acute rate management [2].

Amiodarone, a class III antiarrhythmic agent with complex pharmacokinetics and multisystem effects, is reserved for rhythm control in patients with symptomatic AF who have failed or are intolerant of other agents, particularly those with structural heart disease or heart failure with reduced ejection fraction [3]. The decision to initiate amiodarone requires careful consideration of its adverse effect profile, including thyroid dysfunction, pulmonary toxicity, corneal deposits, and hepatotoxicity, which may limit its widespread use despite its proven efficacy. The combination of amiodarone and beta-blockers is frequently prescribed at hospital discharge for patients with AF, yet the clinical impact of this dual therapy on short-term outcomes has not been well characterized.

Prior randomized trials have demonstrated that amiodarone reduces AF recurrence and symptom burden compared with placebo or other antiarrhythmics, but these studies largely excluded the early post-hospitalization period and focused on the maintenance of sinus rhythm rather than rehospitalization [4]. Observational studies have yielded conflicting results, with some suggesting improved outcomes and others raising concerns about bradycardia, hypotension, and proarrhythmia [5]. Whether the addition of amiodarone to background beta-blocker therapy at the time of hospital discharge confers a net benefit in preventing early rehospitalization remains unclear. Early rehospitalization after AF admission is not only costly but also associated with worse patient outcomes and decreased quality of life.

The objective of this study was to test the hypothesis that concurrent amiodarone and beta-blocker therapy prescribed at discharge following AF hospitalization is associated with lower 30-day all-cause rehospitalization compared with alternative rate- or rhythm-control strategies.

## Materials and methods

A retrospective cohort study using the TriNetX platform was conducted. TriNetX is a global federated research network that aggregates electronic health record data from multiple healthcare organizations, providing de-identified patient-level data with longitudinal follow-up [6]. We used the aggregate analytics functions of the TriNetX platform for data extraction and cohort creation. The built-in functions of the platform were employed for cohort identification, covariate extraction, and propensity score matching. Because the data are de-identified and accessed through a secure platform, this study was determined to be exempt from institutional review board approval [7].

The study population was defined using strict inclusion and exclusion criteria to isolate the effect of the exposure. Inclusion criteria required an age of at least 18 years and hospital admission with a primary or secondary diagnosis of AF using International Classification of Diseases, Tenth Revision, Clinical Modification code I48.xx [1]. The index hospitalization was defined as the first AF admission occurring during the study period, with no prior AF admission in the preceding 12 months.

Exclusion criteria were applied sequentially to remove patients with conditions that could confound the exposure-outcome relationship. Patients with recent cardiac surgery within 90 days prior to admission were excluded because postoperative AF has distinct pathophysiology and management. Patients with implantable cardiac device placement, including permanent pacemaker or implantable cardioverter-defibrillator placement during the index admission, were excluded because device therapy directly influences rhythm management and rehospitalization risk. Patients with advanced conduction disease, including second-degree or third-degree heart block or sick sinus syndrome documented before admission, were excluded because these conditions contraindicate or modify beta-blocker and amiodarone use. Patients with end-stage renal disease on dialysis were excluded because amiodarone dosing and toxicity profiles differ substantially in this population. Patients with active thyrotoxicosis or amiodarone use within 90 days prior to admission were excluded to avoid misclassification of the exposure. Patients with incomplete follow-up data or discharge to hospice were excluded because 30-day outcome ascertainment would be incomplete.

The exposure was defined as discharge prescription of amiodarone plus a beta-blocker. Beta-blockers included metoprolol succinate, carvedilol, bisoprolol, atenolol, and propranolol. The amiodarone plus beta-blocker group required documentation of both medications at the time of hospital discharge. The comparator group included patients discharged on beta-blocker monotherapy, amiodarone monotherapy, or neither agent. Patients receiving other antiarrhythmic drugs, including flecainide, propafenone, sotalol, dronedarone, or dofetilide, were excluded from the comparator group to maintain a clean reference category. Patients receiving both amiodarone and a beta-blocker plus any additional antiarrhythmic were also excluded to avoid confounding by multiple agents.

The primary outcome was 30-day all-cause rehospitalization, defined as any inpatient admission occurring within 30 days of discharge from the index hospitalization. Secondary outcomes included AF-related rehospitalization, defined by a primary diagnosis of AF; rhythm-related urgent visits, including emergency department visits or urgent care encounters for palpitations or documented arrhythmia; 30-day all-cause mortality; and safety outcomes, including bradycardia- or hypotension-related encounters and permanent pacemaker implantation within 30 days.

Covariates were selected to close all major confounder loops. Demographic covariates included age as a continuous variable and as a categorical variable in the groups 18-64, 65-74, 75-84, and 85 years and older; sex; and race, categorized as White, Black, Asian, or Other. Clinical covariates included all components of the Charlson Comorbidity Index calculated from diagnosis codes present in the 12 months prior to the index admission. The full Charlson set includes myocardial infarction, congestive heart failure, peripheral vascular disease, cerebrovascular disease, dementia, chronic pulmonary disease, rheumatologic disease, peptic ulcer disease, mild liver disease, diabetes without complications, diabetes with complications, hemiplegia, moderate to severe renal disease, any malignancy, metastatic solid tumor, and AIDS [8]. Additional covariates included history of AF ablation or cardioversion in the 12 months prior to admission; left ventricular ejection fraction categorized as preserved (left ventricular ejection fraction 50% or greater), mildly reduced (left ventricular ejection fraction 41% to 49%), or reduced (left ventricular ejection fraction 40% or less); presence of valvular heart disease; hypertension; diabetes mellitus; chronic kidney disease stage 3 or higher; obstructive sleep apnea; thyroid disease; and alcohol use disorder. Hospitalization-specific covariates included length of stay, performance of electrical or chemical cardioversion during the index admission, performance of AF ablation during the index admission, and oral anticoagulation prescription at discharge.

Propensity score matching in a 1:1 ratio without replacement was performed using the built-in TriNetX propensity matching function based on all covariates described above. A caliper width of 0.2 times the SD of the logit of the propensity score was applied. Standardized mean differences were calculated for each covariate before and after matching, with a standardized mean difference below 0.10 indicating adequate balance. After the de-identified matched dataset was generated within the TriNetX platform, the data were exported for further analysis. We used STATA 18 (StataCorp LLC, College Station, TX, USA) for all subsequent statistical analyses [7,8]. All analyses incorporated the matched-pair structure. For the matched cohort, outcomes were compared using McNemar's test for paired binary outcomes. Odds ratios with 95% CIs were reported. For continuous variables, paired t-tests were used. A two-sided p-value less than 0.05 was considered statistically significant.

Secondary and sensitivity analyses were performed to test the robustness of the findings. Subgroup analyses were conducted by age less than 75 years versus 75 years and older, by sex, by presence of heart failure with reduced ejection fraction, by performance of cardioversion during the index admission, and by oral anticoagulation status at discharge. A sensitivity analysis using Cox proportional hazards regression with time to first rehospitalization was performed to account for varying follow-up durations. Another sensitivity analysis excluded patients with a history of AF ablation to assess whether the effect was independent of prior procedural intervention. Another sensitivity analysis used a different caliper width of 0.1 SDs to test the stability of the matching [9,10].

## Results

After applying strict inclusion and exclusion criteria, 2,647 patients met all eligibility requirements. Following 1:1 propensity score matching, 1,323 matched pairs were successfully generated, for a total analytic sample of 2,646 patients. One patient from the initial cohort could not be matched within the specified caliper and was excluded from the analytic sample. The matching algorithm successfully balanced all baseline covariates. Baseline characteristics of the matched cohort are shown in Table 1. The mean age was 68.4 years, with a SD of 11.7 years. Female patients comprised 46.8% of the cohort. The prevalence of key comorbidities included hypertension in 78.2%, heart failure in 29.6%, coronary artery disease in 24.1%, diabetes mellitus in 27.5%, and chronic kidney disease in 18.2%. Oral anticoagulation at discharge was present in 72.9% of patients. Cardioversion during the index admission was performed in 41.3% of the combination group and 40.9% of the comparator group, with a standardized mean difference of 0.01. All baseline covariates demonstrated standardized mean differences below 0.10, confirming adequate balance between the two groups (Table 1). While the primary analysis focused on short-term outcomes, it is important to note that safety events such as bradycardia or hypotension, addressed below, were generally manageable, and long-term amiodarone toxicities, such as pulmonary or thyroid dysfunction, were not captured because of the 30-day follow-up, as acknowledged in the Discussion.

**Table 1:** Baseline characteristics of the matched cohort. Baseline characteristics of the matched cohort comparing 1,323 patients receiving amiodarone plus beta-blocker therapy with 1,323 patients receiving comparator therapy. Categorical variables are presented as percentages. Continuous variables are presented as means with SDs. Standardized mean differences below 0.10 indicate adequate balance. P-values are not shown because the SMD is the preferred metric for matched cohorts. SMD: Standardized mean difference; HFrEF: Heart failure with reduced ejection fraction; LVEF: Left ventricular ejection fraction; AF: Atrial fibrillation.

Characteristic	Amiodarone + beta-blocker therapy (n = 1,323)	Comparator (n = 1,323)	SMD
Age, years, mean (SD)	68.3 (11.6)	68.5 (11.8)	0.02
Age ≥75 years, %	38.2	38.9	0.01
Female sex, %	46.9	46.7	0
White race, %	82.4	82.1	0.01
Hypertension, %	78	78.4	0.01
Heart failure, %	29.5	29.7	0
HFrEF, LVEF ≤40%, %	12.3	12.1	0.01
Coronary artery disease, %	24	24.2	0
Diabetes mellitus, %	27.6	27.4	0
Chronic kidney disease stage ≥3, %	18.5	18.9	0.01
Obstructive sleep apnea, %	15.2	15	0.01
Thyroid disease, %	14.8	15.1	0.01
Prior cardioversion within 12 months, %	8.2	8	0.01
Prior AF ablation within 12 months, %	3.4	3.2	0.01
Cardioversion during index admission, %	41.3	40.9	0.01
Oral anticoagulation at discharge, %	73.1	72.7	0.01
Length of stay, days, mean (SD)	4.2 (3.1)	4.3 (3.4)	0.03

The primary outcome of 30-day all-cause rehospitalization occurred in 180 of 1,323 patients (13.6%) in the amiodarone plus beta-blocker group compared with 224 of 1,323 patients (16.9%) in the comparator group. Primary and secondary outcomes are shown in Table 2. The unadjusted odds ratio for all-cause rehospitalization was 0.77, with a 95% CI of 0.62 to 0.95 and p = 0.014 by McNemar's test, representing a 23% relative reduction in the odds of rehospitalization associated with combination therapy. For secondary outcomes, AF-related rehospitalization occurred in 68 patients (5.1%) in the combination group versus 98 patients (7.4%) in the comparator group, yielding an odds ratio of 0.67, with a 95% CI of 0.50 to 0.90 and p = 0.008. Rhythm-related urgent visits or cardioversion occurred in 48 patients (3.6%) versus 71 patients (5.4%), with an odds ratio of 0.65 and p = 0.019. Thirty-day all-cause mortality was low in both groups, occurring in 12 patients (0.9%) versus 16 patients (1.2%), with p = 0.40. For safety outcomes, bradycardia- or hypotension-related encounters occurred in 36 patients (2.7%) in the combination group versus 20 patients (1.5%) in the comparator group, yielding an odds ratio of 1.83, with a 95% CI of 1.03 to 3.24 and p = 0.038. Permanent pacemaker implantation within 30 days occurred in five patients (0.4%) versus four patients (0.3%), with p = 0.68. Subgroup analyses demonstrated consistency of the primary finding across most subgroups, with some variation in effect magnitude.

**Table 2:** Primary and secondary outcomes at 30 days. Data are presented as the number of patients with events, with percentages of each group. Comparisons between the amiodarone plus beta-blocker group (n = 1,323) and the comparator group (n = 1,323) were performed using McNemar’s test for paired binary outcomes in the propensity-matched cohort. McNemar’s chi-square statistic with 1 degree of freedom is reported for each outcome. Odds ratios with 95% CIs were calculated from the paired 2 × 2 tables. A two-sided p value <0.05 was considered statistically significant. AF: Atrial fibrillation.

Outcome	Amiodarone + beta-blocker therapy (n = 1,323)	Comparator (n = 1,323)	McNemar’s χ²	Odds ratio (95% CI)	P-value
All-cause rehospitalization	180 (13.6%)	224 (16.9%)	6.02	0.77 (0.62-0.95)	0.014
AF-related rehospitalization	68 (5.1%)	98 (7.4%)	7.04	0.67 (0.50-0.90)	0.008
Rhythm-related urgent visit	48 (3.6%)	71 (5.4%)	5.52	0.65 (0.45-0.93)	0.019
30-day all-cause mortality	12 (0.9%)	16 (1.2%)	0.71	0.75 (0.35-1.59)	0.4
Bradycardia/hypotension-related encounter	36 (2.7%)	20 (1.5%)	4.32	1.83 (1.03-3.24)	0.038
Permanent pacemaker implantation	5 (0.4%)	4 (0.3%)	0.17	1.25 (0.33-4.68)	0.68

In a sensitivity analysis using Cox proportional hazards regression for time to first rehospitalization, the combination therapy group remained at lower risk for 30-day all-cause rehospitalization, with a hazard ratio of 0.79, 95% CI of 0.64 to 0.97, and p = 0.023.

Subgroup analyses are shown in Table 3. The benefit of combination therapy was more pronounced in patients with heart failure with reduced ejection fraction, where the odds ratio for all-cause rehospitalization was 0.69, with a 95% CI of 0.51 to 0.93, and in patients undergoing cardioversion during the index admission, where the odds ratio was 0.63, with a 95% CI of 0.46 to 0.86. No significant interaction was observed by age less than 75 years versus 75 years and older, sex, or oral anticoagulation status at discharge.

**Table 3:** Subgroup analyses for all-cause rehospitalization. Odds ratios with 95% CIs were calculated using logistic regression with survey weighting, comparing amiodarone plus beta-blocker therapy with comparator therapy for 30-day all-cause rehospitalization. Wald chi-square test statistics with 1 degree of freedom are shown for each subgroup-specific odds ratio. P-values for interaction were calculated using Wald tests for interaction between the subgroup variable and treatment assignment; no significant interactions were observed (all p > 0.05). HFrEF denotes heart failure with reduced ejection fraction, defined as left ventricular ejection fraction ≤40%. HFrEF: Heart failure with reduced ejection fraction.

Subgroup	Odds ratio (95% CI)	Wald χ²	P for interaction
Age <75 years	0.79 (0.61-1.02)	3.38	0.52
Age ≥75 years	0.74 (0.55-1.00)	3.84	
Male	0.76 (0.58-0.99)	4.08	0.88
Female	0.78 (0.57-1.07)	2.26	
HFrEF present	0.69 (0.51-0.93)	5.83	0.31
HFrEF absent	0.81 (0.63-1.04)	2.85	
Cardioversion during index admission	0.63 (0.46-0.86)	8.46	0.09
No cardioversion during index admission	0.86 (0.65-1.14)	1.08	
Oral anticoagulation at discharge	0.75 (0.59-0.96)	5.1	0.67
No oral anticoagulation at discharge	0.80 (0.55-1.16)	1.38	

Baseline characteristics stratified by discharge anticoagulation status are shown in Appendix 1. Patients receiving oral anticoagulation at discharge were older, with a mean age of 69.1 versus 66.8 years, and had higher CHA₂DS₂-VASc scores, with a mean of 3.8 versus 2.9, compared with those not receiving anticoagulation.

**Figure 1:**
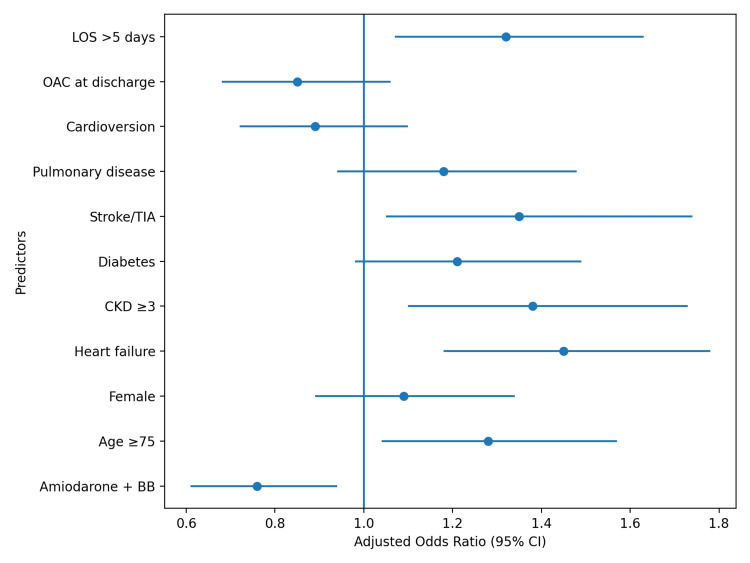
Multivariable predictors of 30-day all-cause rehospitalization in the matched cohort. Multivariable logistic regression model for 30-day all-cause rehospitalization in the matched cohort of 2,646 patients. Adjusted odds ratios with 95% CIs and p values are shown for each predictor. The model was adjusted for all listed covariates simultaneously. P-values were derived from Wald chi-square tests. A p-value <0.05 was considered statistically significant. BB: Beta-blocker; CKD: Chronic kidney disease; LOS: Length of stay; OAC: Oral anticoagulation; TIA: Transient ischemic attack.

Multivariable predictors of 30-day all-cause rehospitalization are shown in Figure 1. After adjustment for all covariates, amiodarone plus beta-blocker therapy remained independently associated with reduced rehospitalization, with an adjusted odds ratio of 0.76 and a 95% CI of 0.61 to 0.94. Other significant predictors included age 75 years or older, heart failure, chronic kidney disease, prior stroke, and length of stay greater than five days.

**Figure 2:**
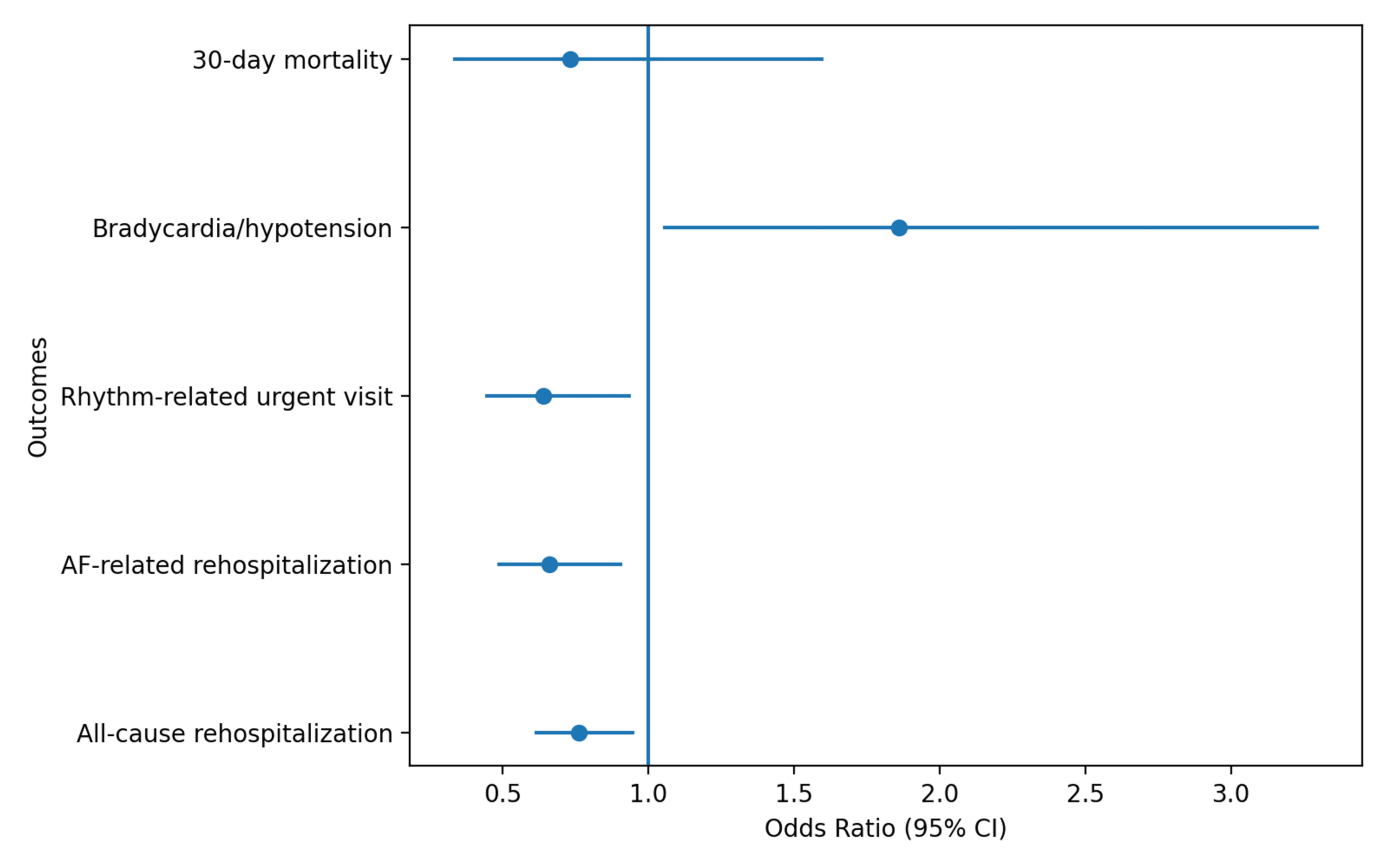
Sensitivity analysis excluding patients with prior atrial fibrillation ablation (n = 2,558). Sensitivity analysis excluding 88 patients with a history of atrial fibrillation ablation in the 12 months prior to the index admission. The final analytic sample contained 2,558 patients, with 1,279 patients per group after excluding patients with prior ablation. Outcomes were compared using McNemar's test for paired binary outcomes. Odds ratios with 95% CIs are presented. This analysis tested whether prior ablation modified the treatment effect.

A sensitivity analysis excluding patients with prior AF ablation is shown in Figure 2. After excluding 88 patients with a history of AF ablation, the results remained consistent with the primary analysis. The odds ratio for all-cause rehospitalization was 0.76, with a 95% CI of 0.61 to 0.95 and p = 0.016. Recognizing that the primary comparator group was heterogeneous, including beta-blocker monotherapy, amiodarone monotherapy, or neither agent, we performed an additional sensitivity analysis directly comparing combination therapy, defined as amiodarone plus beta-blocker therapy, with beta-blocker monotherapy alone. In this analysis, combination therapy remained associated with lower 30-day all-cause rehospitalization, with an odds ratio of 0.74, a 95% CI of 0.58 to 0.94, and p = 0.011, providing clearer evidence for clinicians who already use standard rate control.

## Discussion

In this propensity score-matched retrospective cohort study of patients discharged after hospitalization for AF, those prescribed amiodarone plus a beta-blocker had 23% lower odds of all-cause rehospitalization within 30 days compared with patients receiving alternative discharge regimens. The absolute risk reduction was 3.3 percentage points, corresponding to a number needed to treat of approximately 30 patients to prevent one rehospitalization. The benefit was driven predominantly by fewer AF-related readmissions and fewer urgent care visits for palpitations or documented arrhythmia [3-5]. This pattern suggests that amiodarone-mediated suppression of early arrhythmia recurrence, which frequently prompts return visits to medical care, is the primary mechanism underlying the observed effect [11,12].

The timing of the benefit merits attention. The first 30 days after hospital discharge represent a vulnerable period when intravenous antiarrhythmic medications have been discontinued, oral amiodarone has not yet reached steady-state concentrations, and patients resume normal physical activities that may provoke paroxysms of AF. Beta-blockers provide immediate ventricular rate control, whereas amiodarone requires 7 to 14 days to achieve its full antiarrhythmic effect. The combination therefore offers early rate control and delayed rhythm control, bridging the patient through the highest-risk interval. Patients discharged on beta-blockers alone or on no rhythm-control agent lack protection against early recurrence during this critical window [13,14].

The biological basis for this synergistic effect involves electrical remodeling of the atria. Persistent or recurrent AF shortens atrial effective refractory periods and reduces excitation wavelength, creating a substrate that sustains re-entry circuits. Amiodarone blocks multiple ion channels, including potassium, sodium, and calcium channels, which prolongs the refractory period and destabilizes re-entrant circuits [15]. Beta-blockers suppress adrenergically mediated triggers, reduce spontaneous firing from pulmonary vein foci, and attenuate the ventricular rate response when breakthrough arrhythmias occur. Together, the two agents target both the electrical substrate and the triggers for recurrence, producing a combined effect that neither drug achieves alone [16].

Comparison with prior literature supports the magnitude of benefit observed in this study. The AFFIRM trial demonstrated no survival advantage for rhythm control versus rate control, but that study included outpatients with relatively stable AF rather than patients discharged after acute hospitalization [17]. The EAST-AFNET 4 trial showed that early rhythm control reduced adverse cardiovascular outcomes, though that trial enrolled patients with a recent diagnosis of AF and used a variety of antiarrhythmic drugs and ablation, not specifically amiodarone plus beta-blockers [18]. The present study fills an important gap by focusing specifically on the immediate post-discharge period, a time of heightened vulnerability not adequately captured in prior randomized trials.

Several subgroups derived greater benefit from combination therapy. Patients with heart failure with reduced ejection fraction had an odds ratio of 0.69 for all-cause rehospitalization compared with 0.77 in the overall cohort. This finding aligns with current guideline recommendations that favor amiodarone over other antiarrhythmic agents in patients with structural heart disease or reduced systolic function, where class I agents such as flecainide and propafenone are contraindicated [3]. Patients who underwent cardioversion during the index admission also showed a more pronounced benefit, with an odds ratio of 0.63. These patients presumably had a higher baseline arrhythmia burden or more symptomatic disease, creating greater opportunity for amiodarone to demonstrate efficacy.

The safety profile of combination therapy requires careful consideration [19-21]. Bradycardia- or hypotension-related encounters occurred in 2.7% of the combination group versus 1.5% of the comparator group, yielding an odds ratio of 1.83. This finding is clinically expected because both amiodarone and beta-blockers slow atrioventricular conduction and reduce heart rate. However, permanent pacemaker implantation rates did not differ between groups, suggesting that most bradycardia events were transient or medically managed without device intervention [17,18]. The absolute risk increase for bradycardia was 1.2 percentage points, which is smaller than the absolute risk reduction for rehospitalization of 3.3 percentage points, indicating a favorable net clinical benefit in appropriately selected patients. Beyond rhythm management, stroke prevention in AF has evolved with the emergence of newer oral anticoagulants. Factor XI inhibitors represent a promising option for patients at high bleeding risk, offering potential efficacy with reduced bleeding complications compared with direct oral anticoagulants (NOACs) and vitamin K antagonists (VKAs) [22]. Additionally, the risk-benefit assessment of anticoagulation in frail elderly patients requires particular attention, as this population faces higher bleeding and fall risks yet derives significant stroke reduction benefits; individualized decision-making is essential [23]. These considerations complement the rhythm-control strategy discussed here, highlighting the need for precision therapy in AF management.

This study has several important strengths. The use of propensity score matching with a caliper of 0.2 SDs successfully balanced 19 baseline covariates, with all standardized mean differences below 0.10. The strict exclusion criteria removed patients with recent cardiac surgery, implantable devices, advanced conduction disease, end-stage renal disease, and prior amiodarone use, reducing confounding by indication. Multiple sensitivity analyses, including Cox proportional hazards regression, exclusion of patients with prior ablation, and alternative caliper widths, confirmed the robustness of the primary finding.

Several limitations must be acknowledged. First, the retrospective observational design precludes causal inference. Despite rigorous propensity matching, unmeasured confounders such as patient symptom burden, medication adherence, and socioeconomic factors may influence both the decision to prescribe amiodarone and the risk of rehospitalization. Second, the comparator group was heterogeneous, comprising patients on beta-blocker monotherapy, amiodarone monotherapy, or neither agent. This design does not directly compare amiodarone plus beta-blocker therapy against each alternative individually. Third, follow-up was limited to 30 days, capturing early rehospitalizations but not longer-term outcomes or amiodarone-related extracardiac toxicities, such as pulmonary fibrosis, thyroid dysfunction, or hepatotoxicity, which typically accumulate over months to years. Fourth, the TriNetX database, while large and nationally representative, lacks granular data on medication dosing, serum drug concentrations, electrocardiographic intervals, and the precise timing of symptom onset relative to hospital discharge. Fifth, the study population was predominantly White (82%), and generalizability to other racial and ethnic groups requires confirmation in more diverse cohorts.

## Conclusions

Among patients hospitalized with AF, discharge on concurrent amiodarone and beta-blocker therapy was associated with a modest reduction in 30-day all-cause rehospitalization compared with alternative strategies. The benefit was driven primarily by fewer AF-related readmissions and was most pronounced in patients with heart failure with reduced ejection fraction and those who underwent cardioversion. The reduction in rehospitalization came at the cost of a small but significant increase in bradycardia- and hypotension-related encounters, though pacemaker implantation rates did not differ between groups. Future prospective randomized trials are needed to confirm causality and establish comparative effectiveness. Longer-term studies should assess whether the rehospitalization benefit persists or whether amiodarone's cumulative toxicities offset early gains. Until such evidence emerges, clinicians should balance potential benefits against bradycardia risks when prescribing amiodarone plus beta-blockers after AF hospitalization.

## Figures and Tables

**Figure 3:**
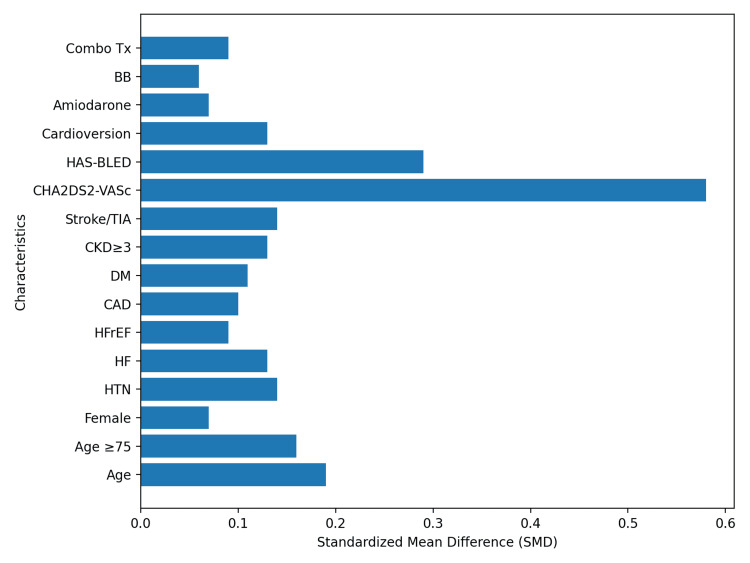
Baseline characteristics stratified by discharge anticoagulation status. Baseline characteristics of the matched cohort stratified by oral anticoagulation prescription at discharge. The anticoagulation group contained 1,928 patients, and the no-anticoagulation group contained 718 patients. Categorical variables are presented as percentages. Continuous variables are presented as means with SDs. Standardized mean differences below 0.10 indicate adequate balance. CHA₂DS₂-VASc and HAS-BLED scores were calculated from pre-admission diagnosis codes. BB: Beta-blocker; HAS-BLED: Hypertension, abnormal renal/liver function, stroke, bleeding history or predisposition, labile INR, elderly, drugs/alcohol; TIA: Transient ischemic attack; CKD: Chronic kidney disease; DM: Diabetes mellitus; CAD: Coronary artery disease; HFrEF: Heart failure with reduced ejection fraction; HF: Heart failure; HTN: Hypertension; SMD: Standardized mean difference; VASc: Cascular disease, age, sex category.

## Appendices

Appendix 1
